# Genomic evolution and dissemination of non-conjugative virulence plasmid of ST65 carbapenem-resistant and hypervirulent *Klebsiella pneumoniae* strains in a Chinese hospital

**DOI:** 10.3389/fcimb.2025.1548300

**Published:** 2025-06-12

**Authors:** Dongxing Tian, Mingqi Zhao, Lihua Liu, Shuhua Lu, Haixin Dong

**Affiliations:** ^1^ Department of Clinical Laboratory, Affiliated Hospital of Jining Medical University, Jining, Shandong, China; ^2^ Institute of traditional Chinese medicine, Postdoctoral Mobile Station of Shandong University of Traditional Chinese Medicine, Jinan, Shandong, China; ^3^ State Key Laboratory of Chemical Biology and Drug Discovery and the Department of Food Science and Nutrition, The Hong Kong Polytechnic University, Kowloon, Hongkong SAR, China; ^4^ Affiliated Hospital of Jining Medical University, Jining, Shandong, China

**Keywords:** Klebsiella pneumoniae, hypervirulence, carbapenem resistance, plasmid transmission, ST65

## Abstract

**Background:**

The global rise in infections caused by hypervirulent and carbapenem-resistant *K. pneumoniae* (CR-hvKp) represents a growing public health threat. This study investigates ST65 CR-hvKp strains, with a focus on their genomic attributes and the mechanisms underlying the transmission of non-conjugative virulence plasmids.

**Methods:**

Two clinical K2-ST65 CR-hvKp isolates (P6 and P10) were identified. Plasmid conjugation experiments were performed to assess the dissemination of the virulence plasmid. Antimicrobial susceptibility testing and virulence assays, including serum resistance, siderophore production, and *G. mellonella* larvae infection models, were used to characterize resistance and virulence phenotypes. Comprehensive bioinformatic analyses were performed to explore genetic evolution.

**Results:**

Genomic analyses showed that both P6 and P10 carry a non-conjugative virulence plasmid, a conjugative untyped KPC plasmid, and a conjugative IncM2 plasmid. These isolates displayed broad-spectrum anti-microbial resistance and multiple virulence phenotypes, although they failed to sustain both hypervirulence and carbapenem resistance over time. The IncM2 plasmid was shown to be essential for the transfer of non-conjugative virulence plasmid. Mechanistic studies highlighted IS*26*-mediated plasmid fusion and the role of IncM2 plasmids in mobilizing non-conjugative virulence plasmids. The resulting transconjugants exhibited multidrug resistance, enhanced capsule production, and increased siderophore production.

**Conclusions:**

This study provides new insights into the genomic dynamics of ST65-CR-hvKp strains and uncovers key mechanisms, such as IS*26*-mediated plasmid fusion and IncM2-mediated mobilization, which facilitate the dissemination of non-conjugative virulence plasmids. Understanding these mechanisms is crucial for developing effective strategies to manage and prevent the spread of these clinically challenging strains.

## 1 Introduction

*Klebsiella. pneumoniae*, a Gram-negative commensal bacterium, colonizes the gastrointestinal tract intestines of patients and the skin of hospital staff (Martin and Bachman, 2018). It is frequently implicated in healthcare-associated infections due to its ability to spread through respiratory droplets and direct contact (Borgmann et al., 2018; Al Bshabshe et al., 2020). The organism’s resistance to multiple antibiotics is largely attributed to horizontal gene transfer via plasmids and other mobile genetic elements, making carbapenem-resistant *K. pneumoniae* (CRKP) a major clinical concern (Tamma et al., 2023). Beyond hospital settings, *K. pneumoniae* is also responsible for community-acquired infections such as liver abscesses and endophthalmitis, even in otherwise healthy individuals (Choby et al., 2020). These infections are often associated with a hypermucoviscous phenotype characteristic of hypervirulent *K. pneumoniae* (hvKp) strains (Choby et al., 2020). The CG65 clone is recognized as a hypervirulent lineage enriched with virulence plasmids, including *iuc, iro, rmpA, and rmpA2*, but typically lacking significant antibiotic resistance determinants (Wyres et al., 2019). These strains commonly carry a conserved 224 Kb virulence plasmid and infrequently acquire additional plasmids, resulting in low pan-genome diversity (Lam et al., 2018; Wyres et al., 2019).

Reports of clinical isolates exhibiting both hypervirulence and carbapenem resistance are on the rise (Yang et al., 2022; Pu et al., 2023; Guo et al., 2024). A previous study (Tian et al., 2022) categorized these dual-threat strains into two evolutionary pathways: 1) CR-hvKp, where hvKp strains acquire resistance plasmids, and 2) hv-CRKP, where CRKP strains acquire virulence plasmids. Current evidence suggests the hv-CRKP pathway is more prevalent (Han et al., 2022; Yang et al., 2022), likely due to the facilitative role of *bla*_KPC_-bearing IncFII plasmids that can co-mobilize non-conjugative virulence plasmids through shared *oriT* sequences (Xu et al., 2021; Tian et al., 2022). Conversely, CR-hvKp strains must acquire resistance elements despite innate barriers intrinsic to hvKp, including hypercapsule production and active CRISPR-Cas systems, which impair plasmid acquisition and maintenance (Lam et al., 2018; Zhou et al., 2020). Notably, acquisition of KPC-encoding plasmids has been shown to attenuate capsule synthesis in hvKp strains, reflecting an evolutionary trade-off between resistance and virulence (Tian et al., 2022). Due to the limited number of clinical CR-hvKp isolates, the mechanisms of plasmid acquisition, maintenance, and transmission in these strains remain poorly understood. Therefore, it is necessary to investigate the prevalence and dissemination of hv-CRKP strains in hospitals.

This study investigates two ST65 K2-serotype CR-hvKp isolates, providing insights into their genomic architecture and unveiling novel mechanisms of plasmid mobilization that facilitate the simultaneous spread of hypervirulence and carbapenem resistance in clinical settings.

## 2 Materials and methods

### 2.1 Strains and definitions

Two isolates, P6 and P10, were identified as K2 serotype and sequence type 65 (ST65), both carrying the *bla*_KPC-2_ and *iucA* genes. Bacterial species identification was confirmed using MALDI-TOF MS (Biotyper Microflex, Bruker Daltonics, Bremen, Germany).

Additionally, 1478 complete *Klebsiella* genomes (retrieved on 31 December 2022) were downloaded from the NCBI database (https://www.ncbi.nlm.nih.gov/datasets/genome/) (Datasheet 1). Sequence types, K loci, virulence and antibiotic resistance genes, and corresponding scores were analyzed using *Kleborate* (http://github.com/katholt/Kleborate). Resistance scores were calculated as follows: 0 = no ESBL or carbapenemase; 1 = ESBL without carbapenemase (regardless of colistin resistance); 2 = carbapenemase without colistin resistance (regardless of ESBL); 3 = carbapenemase with colistin resistance (regardless of ESBL). Virulence scores were calculated as follows: 0 = none present; 1 = yersiniabactin only; 2 = colibactin without aerobactin (regardless of yersiniabactin; however, ybt is almost always co-present with clb); 3 = aerobactin only; 4 = aerobactin and yersiniabactin without colibactin; and 5 = all three present. CRKP was defined as carrying carbapenem resistance genes (with resistance scores exceeding 2). HvKp was defined by the presence of virulence-associated genes (virulence scores exceeding 3). Convergent *K. pneumoniae* was defined as carrying both carbapenem resistance genes and virulence-associated genes (resistance score more than 2 and virulence score more than 3).

### 2.2 Phylogenetic analysis

To explore the evolutionary relationship between P6, P10 and nine ST65 genomes from NCBI, a phylogenetic tree was constructed based on whole genome SNPs using the CSI Phylogeny 1.4 web-based program. Parameters included a minimum distance of 10 bp, a minimum of 10x depth, 10% breadth coverage, mapping quality > 30, and SNP quality > 25 (Kaas et al., 2014). SNPs were called by BWA v. 0.7.2 and SAMtools v. 0.1.18, leaving out all SNPs in a 10-base vicinity of each other. CSI phylogeny returns a newick file for tree visualization (Jensen et al., 2021). P6 strain served as the reference genome. All genome and sequence assemblies were uploaded in a FASTA format. The tree was visualized and annotated by Evolview v3 (https://www.evolgenius.info/evolview).

### 2.3 Antimicrobial susceptibility tests

Antimicrobial susceptibility testing was performed for P6, P10 and their transconjugants against 16 antibiotics using the broth microdilution method. Minimum inhibitory concentration (MIC) were determined following Clinical and Laboratory Standards Institute guidelines (CLSI-M100), with *Escherichia coli* ATCC 25922 used as a quality control.

### 2.4 Plasmid conjugation experiments

Plasmid conjugation was conducted using sodium azide-resistant *E. coli* J53 as the recipient and P6 and P10 strains as donors. Equal volumes of donor and recipient logarithmic-phase cultures were mixed and incubated overnight at 37°C to facilitate conjugation. Transconjugants were selected on agar containing sodium azide (80 μg/ml) and potassium tellurite (1.5 μg/ml). The virulence plasmid carries potassium tellurite resistance genes. PCR assays targeting *iucA* (encoding aerobactin synthetase), and *oqxA* (belonging to the RND family) were used to confirm the transfer of non-conjugative virulence plasmid to *E. coli* J53. The *oqxA* gene is chromosomal in the donor strains P6 or P10 but absent in the recipient strain J53, allowing for genotype-based distinction. Consequently, the donor strains exhibit an *iucA*-positive and *oqxA*-positive genotype, the recipient strain shows an *iucA*-negative and *oqxA*-negative genotype, and the transconjugants are *iucA*-positive but *oqxA*-negative.

A second-round plasmid conjugation was performed using rifampicin-resistant *E. coli* C600 as the recipient and the previous transconjugants as donors. Transconjugants were selected on agar containing rifampicin (200 μg/ml) and potassium tellurite (1.5 μg/ml). PCR assays targeting *iucA*, *and YeeU* (type IV toxin-antitoxin system) were used to confirm the success of transconjugation. The *YeeU* gene is chromosomal in the recipient strain C600 but absent in the donor strains. Consequently, the donor strains exhibit an *iucA*-positive and *YeeU*-negative genotype, the recipient strain shows an *iucA*-negative and *YeeU*-positive genotype, and the transconjugants are *iucA*-positive but *YeeU*-positive. The primers are listed in **Supplementary Table S1**. Plasmids in transconjugants were visualized using S1 nuclease-Pulsed-Field Gel Electrophoresis (S1-PFGE) (Tian et al., 2020).

### 2.5 Plasmid stability

Plasmid stability was tested as previously described (Zhou et al., 2020). Briefly, P6 and P10 were cultured overnight at 37°C in LB medium with meropenem (1 µg/ml) and potassium tellurite (1.5 µg/ml), then serially passaged 50 times (12 hours/passage) in antibiotic-free LB. The plasmid loss rate was calculated per generation (Zhou et al., 2020).

### 2.6 Whole genome sequencing

P6, P10, and their transconjugants were sequenced using the Illumina NovaSeq and MinION platforms. Raw data quality control was performed with FastQC software (v. 0.11.8, http://www.bioinformatics.babraham.ac.uk/projects/fastqc/). *De novo* genome assembly was performed using HGAP and CANU with default settings (Chin et al., 2016; Koren et al., 2017), and the assembly correction was performed using Pilon v1.22 (Walker et al., 2014). Circular plasmid maps for comparative analysis were generated using the BLAST Ring Image Generator (BRIG) (http://sourceforge.net/projects/brig). Prediction of *oriT* sequences and transfer-associated elements was carried out using oriTfinder’s default parameters (https://bioinfo-mml.sjtu.edu.cn/oriTfinder/).

### 2.7 Plasmid construction and targeted plasmid elimination

The pCasRif plasmid, containing the *arr-3* gene for rifampin resistance, was constructed as previously described (Hao et al., 2020). Single guide RNAs (sgRNA) targeting the replicons of the pP6P3 plasmid and pP6Inc were designed using the IDT CRISPR design tool (https://eu.idtdna.com/site/order/designtool/index/CRISPR_CUSTOM). The sgRNA fragments were amplified using pCascure as a template, and primer sequences are listed in **Supplementary Table S1**. The PCR products were cloned into XbaI- and SpeI-digested pCasCure to generate the targeted constructs pCasCure-IncM and pCasCure-P3. These constructs were introduced into *K. pneumoniae* P6 by electroporation, and transformants were selected on LB agar plates supplemented with rifampin (200 μg/ml). Successful plasmid transfer was confirmed by PCR. To eliminate the target plasmids, the transformants were cultured LB broth containing 0.1% arabinose and 200 μg/ml rifampin for 6 hours at 37 °C with aeration. Cultures were then serially diluted and plated on LB agar plates, and plasmid loss was confirmed by PCR. The resulting *K. pneumoniae* P6-P3cured and P6-IncMcured strains were co-cultured with *E. coli* J53 to assess the conjugative transfer potential of the virulence plasmid.

### 2.8 Virulence-associated experiments

Virulence was evaluated for strains P6, P10, and transconjugants using multiple assays. The *K. pneumoniae* RJF293 strain (Accession: CP014008.1), a confirmed hypervirulent K2-ST65 strain (Wang et al., 2018), was used as a positive control, while the ST11 strain HS11286 (Accession: CP003200.1) served as a negative control. Assays for viscosity, capsular polysaccharide (CPS) production, siderophore production, and *G. mellonella* larvae infection were performed as described previously (Tian et al., 2022). Briefly, overnight bacteria cultures were centrifuged at 10000g for 30 seconds, and the optical density (OD600) of the supernatants was measured to assess viscosity. CPS production was quantified by measuring uronic acid content. Siderophore production was assessed using the Chrome Azurol S (CAS) Assay. Serum resistance was performed according to a previous study (Sahly et al., 2004). Bacterial cultures in mid-log phase (adjusted to OD600 = 0.5) were mixed with normal human serum at a 1:3 ratio and incubated at 37°C for 3 hours. Both self-controls and blank controls were included to validate the assay. Serum resistance was determined by plotting the average survival percentage of each strain over the incubation period.

### 2.9 Statistical analysis

All statistical analyses were conducted using GraphPad Prism 9 (GraphPad Software, San Diego, CA, USA). Student’s t-test was used for comparing means, and the log-rank test was employed for survival curve analysis.

## 3 Results

### 3.1 Identification of ST65 hypervirulent and carbapenem-resistant *K. pneumoniae* strains

**Figure 1:**
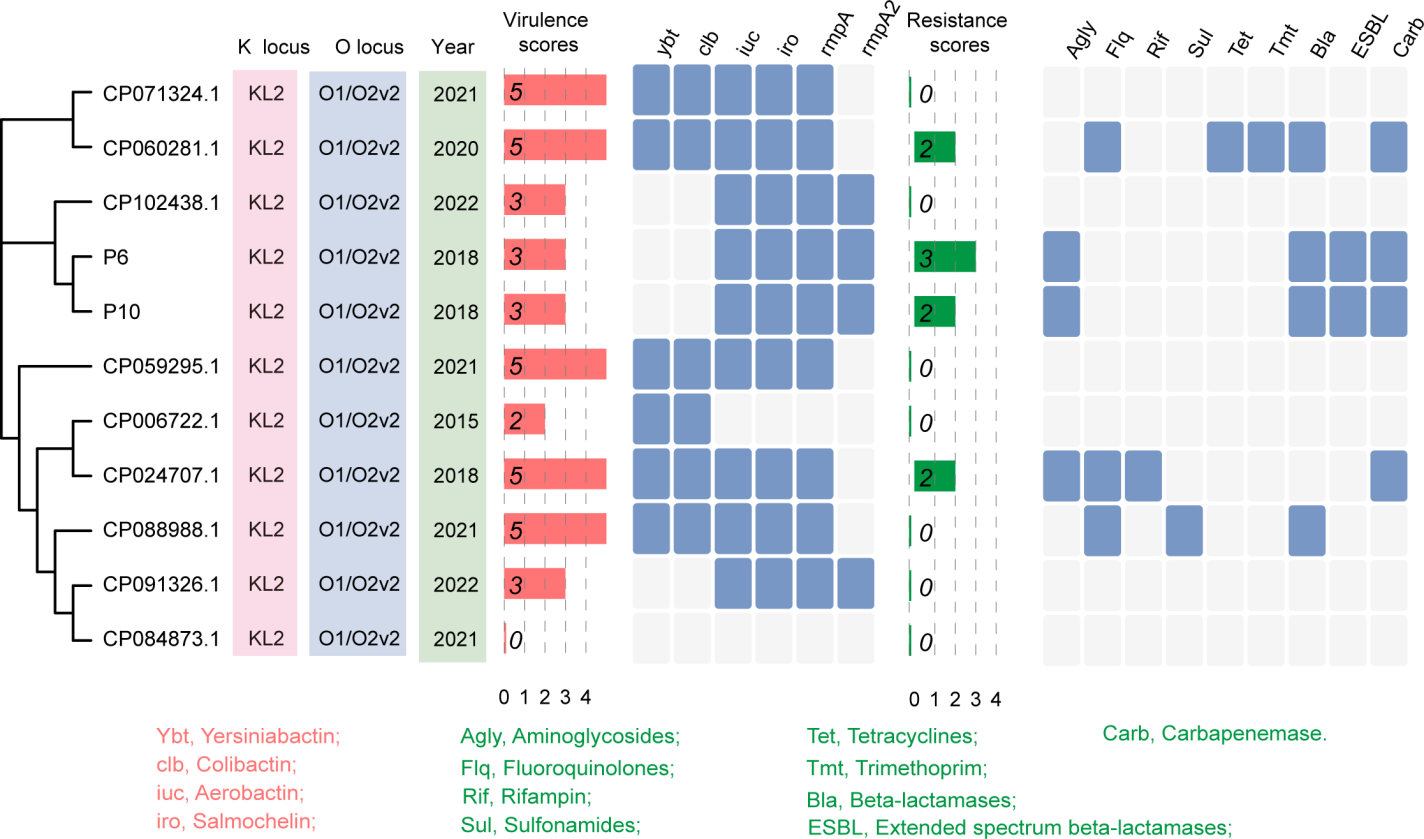
Identification of ST65 hypervirulent and carbapenem-resistant *K. pneumoniae.* Phylogenetic analysis and basic characteristics of ST65 *K. pneumoniae* strains. The phylogenetic tree was constructed based on the core genome sequences of P6 and P10 strains (isolated in this study), and nine ST65 *K. pneumoniae* strains retrieved from the GenBank database.

Carbapenem-resistant and hypervirulent *K. pneumoniae* strains P6 and P10 were isolated from sputum cultures of ICU and neurosurgery patients on April 13 and June 11, 2020, respectively. Notably, the hospital stays of the two patients did not overlap. To investigate the genetic background of these isolates, we sequenced and assembled their genomes. A SNP-based phylogenetic analysis showed that P6 and P10 were almost identical, differing by only one SNP, and shared the same profiles of virulence and resistance genes (**Figure 1**). We compared two isolates with nine ST65 genomes from NCBI to further characterize their virulence and resistance. Most ST65 strains exhibited high virulence scores and low resistance scores (**Figure 1**). In contrast, P6 and P10 displayed both high virulence and high resistance scores (**Figure 1**), indicating the emergence of ST65 strains with a convergent resistance-virulence phenotype.

### 3.2 Mobilization of non-conjugative virulence plasmids

Both P6 and P10 harbored four plasmids: (i) a KPC plasmid, (ii) a virulence plasmid, (iii) an IncM2 plasmid carrying *aac(3)-IId*, *bla*_TEM-1_, *bla*_CTX-M-15_, and (iv) a ColRNAI plasmid (**Table 1**). Except for IncM2, the KPC, virulence, and ColRNAI plasmids of P6 and P10 were almost identical (**Supplementary Figure S1**). The IncM2 plasmid in P6 has indel and a rearrangement difference compared to the IncM2 plasmid of P10, producing two copies of *aac(3)-IId* in P6. The *bla*_KPC_ gene was located on an untyped plasmid (P3). Conjugation experiments confirmed that both the KPC and IncM2 plasmids were transferable (**Supplementary Figure S2**), indicating potential for mobilizing non-conjugative plasmids. The 228-kb virulence plasmid (IncFIB/IncHI1B type) lacked intact transfer machinery such as Type IV Secretion System (T4SS).

**Table 1:** The genomic characteristics of ST65 hypervirulent and carbapenem-resistant *K. pneumoniae* strains in this study.

Characteristics	P6				P10			
Chromosome (Kb)	5.2				5.2			
Plasmids	pP6Vir	pP6Inc	pP6P3	pP6Col	pP10Vir	pP10Inc	pP10P3	pP6Col
Accession no.	CP139687.1	CP139688.1	CP139689.1	CP139690.1	CP139682.1	CP139683.1	CP139684.1	CP139685.1
Length (bp)	228286	86070	42848	20112	228285	74500	42848	20112
Incompatibility group	IncFIB_K_/IncHI1B	IncM2	untypeable	ColRNAI	IncFIB_K_/IncHI1B	IncM2	untypeable	ColRNAI
Conjugal transfer elements								
oriT	+^a^	+	+	–	+	+	+	–
Relaxase	-^b^	+	+	–	–	+	+	–
T4CP	+	+	+	–	+	+	+	–
T4SS	–	+	+	–	–	+	+	–
Resistance factors	–	*aac(3)-IId* *bla*_TEM-1_ *bla*_CTX-M-15_	*bla*_KPC-2_	–	–	*aac(3)-IId* *bla*_TEM-1_ *bla*_CTX-M-15_	*bla*_KPC-2_	–
Virulence factors	*iucABCD-iutA;* *iroBCD-iroN* *rmpA* *rmpA2*	–	–		*iucABCD-iutA;* *iroBCD-iroN* *rmpA* *rmpA2*	–		

a+, have such information.

b-, no such information.

**Figure 2:**
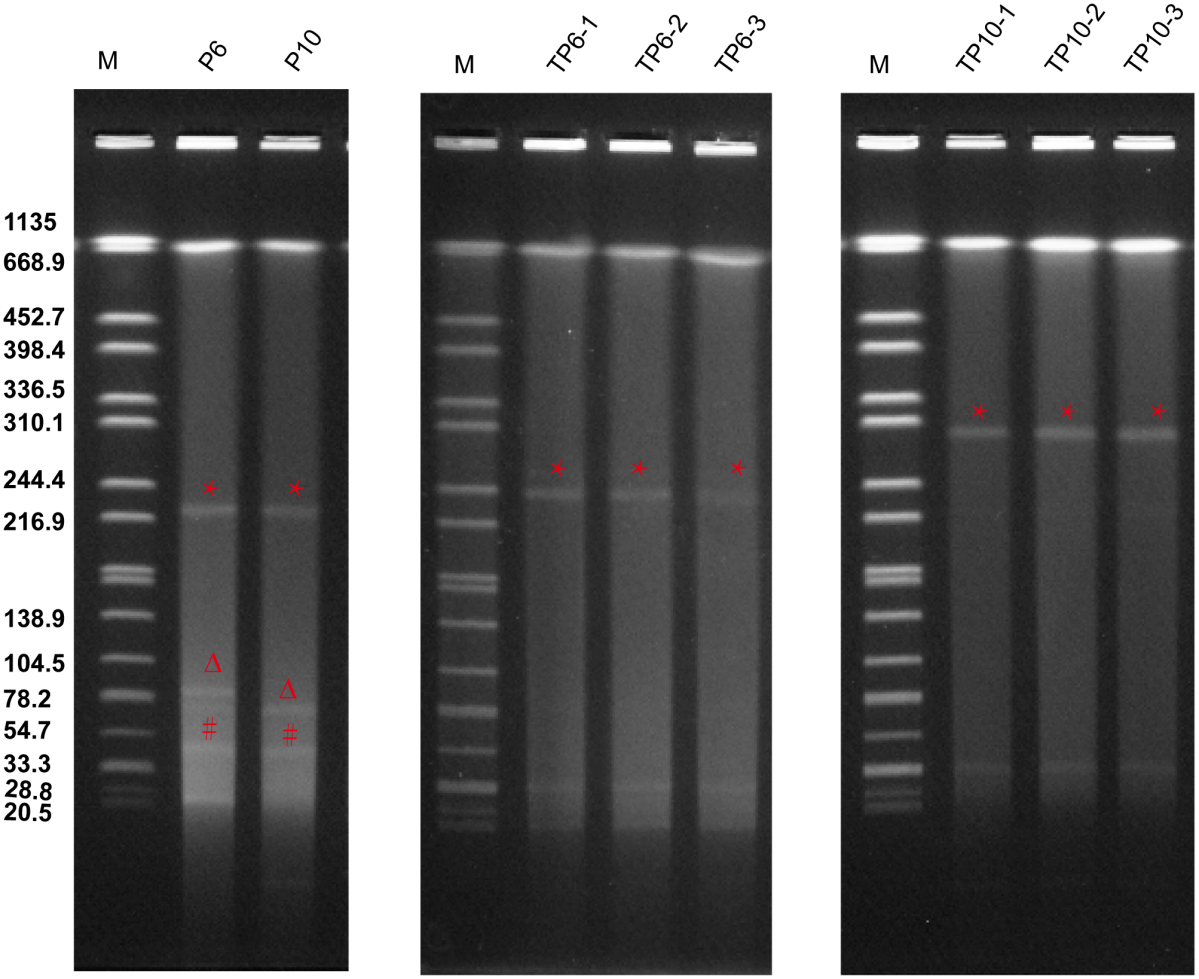
Plasmid profiles of P6, P10 and their transconjugants. S1-PFGE analysis was performed on three TP6 transconjugants (TP6-1, TP6-2, and TP6-3) and three TP10 transconjugants (TP10-1, TP10-2, and TP10-3) that were randomly selected to perform. * indicate virulence plasmids (pVir), Δ indicate IncM2 plasmids (pInc), # indicate KPC plasmids (pP3).

To investigate if the virulence plasmids could still be mobilized, we co-cultured ST65-CR-hvKp with *E. coli* J53. Both P6 and P10 successfully transferred their virulence plasmids at conjugation frequencies of 6.5 ± 3.4×10^-6^ and 4.6 ± 2.3×10^-6^, respectively (**Figure 2**). However, the plasmid profiles of transconjugants differed from their parental strains. Both P6 transconjugants (TP6) and P10 transconjugants (TP10) acquired an identical 33 Kb plasmid p3 (**Figure 2**). We aligned all plasmids from the donor and recipient (**Supplementary Figure S3**), but their origin was unclear. Additionally, TP6 strains harbored a 240 Kb-plasmid, while P10 strains contained a 300 Kb plasmid (**Figure 2**).

### 3.3 IS*26*-mediated the plasmid fusion

**Figure 3:**
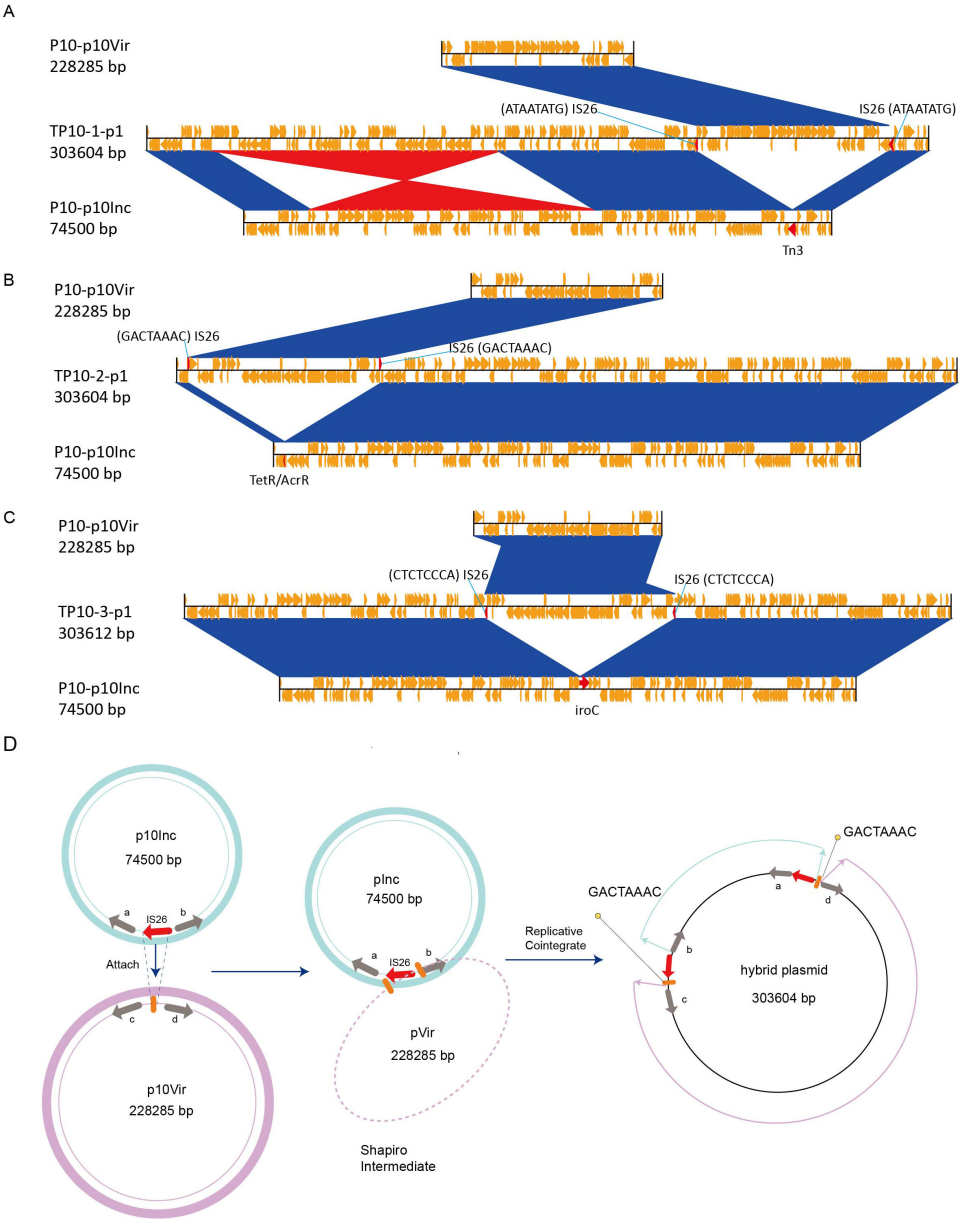
Comparative genome analysis of hybrid plasmid (TP10-pVir) and proposed mechanism of plasmid fusion. **(A-C)** Comparative genome analysis of hybrid plasmid identified in TP10-1, TP10-2, and TP10-3, respectively. **(D)** Proposed model of IS*26*-mediated plasmid fusion using TP10-2-p1 as an example. Red arrows represent IS*26* elements, and orange bold string represent the 8 bp target site duplication (GACTAAAC).

To explore plasmid evolution in the transconjugants, three clones each from TP6 and TP10 were randomly selected for sequencing and assembly (**Supplementary Table S2**). Comparative genomics revealed that the 303 kb hybrid plasmid in TP10 formed via fusion of the virulence plasmid (pP10Vir) and the IncM2 plasmid (pP10Inc) at different insertion sites (**Figures 3A-C**). An inversion was also observed during plasmid fusion (**Figure 3A**). Upon closer inspection of the overall structure of the hybrid plasmid, we postulated a plausible pattern of cointegrate formation (**Figure 3D**). IS*26* in pP10Inc targeted a site (e.g., GACTAAAC) pVir, leading to replicative cointegrate formation. The cointegrate plasmid was formed by incorporating pInc upstream of gene D in pVir, resulting in an 8 bp target site duplication (TSD) and acquisition of an extra IS*26* copy. This produced two direct TSD repeats flanking pInc.

Similarly, pVir plasmids in TP6 transconjugants (229105 bp) gained an additional IS*26* insertion compared to the parental pP6Vir (228286 bp), confirming IS*26*-mediated plasmid fusion and recombination as key drivers of plasmid evolution in clinical *K. pneumoniae* strains.

### 3.4 Movement pattern of virulence plasmid

**Figure 4:**
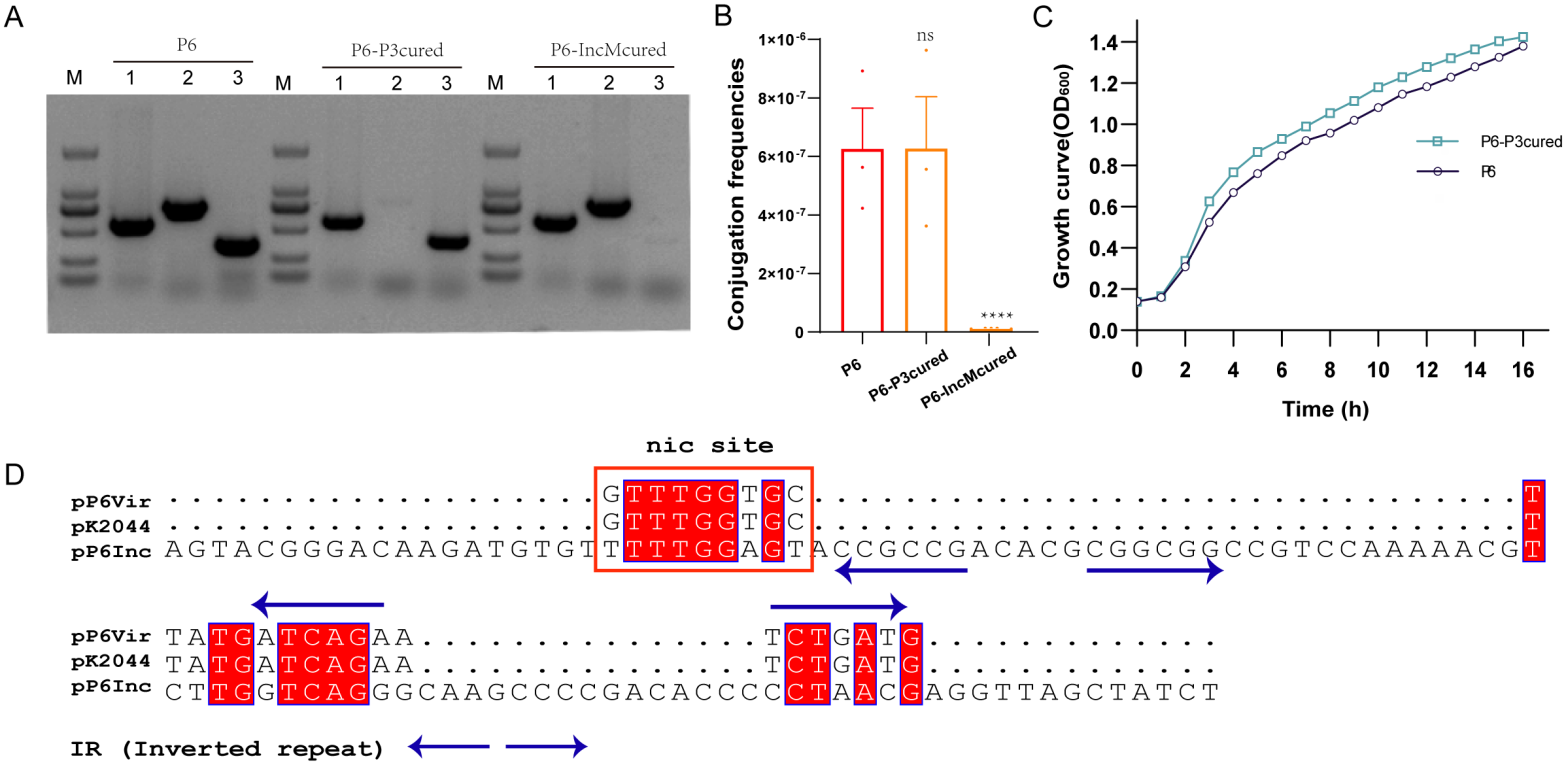
Transmission mechanisms and mobilization of non-conjugative virulence plasmids. **(A)** Elimination of pP3 and pInc plasmids from P6. The P6-P3cured and P6-IncMcured strains were successfully constructed. Lane 1: pVir gene; lane 2: pP3 gene; lane 3: pInc gene. **(B)** Conjugation frequencies of pVir plasmids of P6-P3cured and P6-IncMcured strains. ****P< 0.0001, ns, not significant. **(C)** Growth curves of P6 and P6-P3cured strains. **(D)** Alignment of OriT sequences from pP6Vir, pK2044, and pP6Inc. Multiple sequence alignment was performed using MUSCLE sequence alignment tool. The nic site is highlighted with a red box.

Although fusion with a conjugative plasmid via IS*26* (**Figure 3**) explains virulence plasmid transfer to TP10, it does not account for transfer in TP6. To investigate alternative mobilization mechanisms, we cured P6 of specific plasmids, generating P6-P3cured and P6-IncMcured strains (**Figure 4A**). Conjugation assays showed that pVir remained transferable from P6-P3cured but not from P6-IncMcured (**Figure 4B**), indicating that the IncM2 plasmid, but not the KPC plasmid, mediates pVir mobilization. Additionally, removal of the KPC plasmid imposed fitness costs on P6 compared to the P6-P3cured strain (**Figure 4C**). Sequence analysis revealed the *oriT* sequence of pP6Vir was 100% identical to pK2044 (AP006726.1) and shared 66.7% identity at *nic* site of the IncM2 plasmid (pP6Inc) (**Figure 4D**).

In summary, the non-conjugative virulence plasmid can be transferred either through fusion with or by assistance from the conjugative IncM2 plasmid.

### 3.5 Resistance and virulence phenotypes

**Figure 5:**
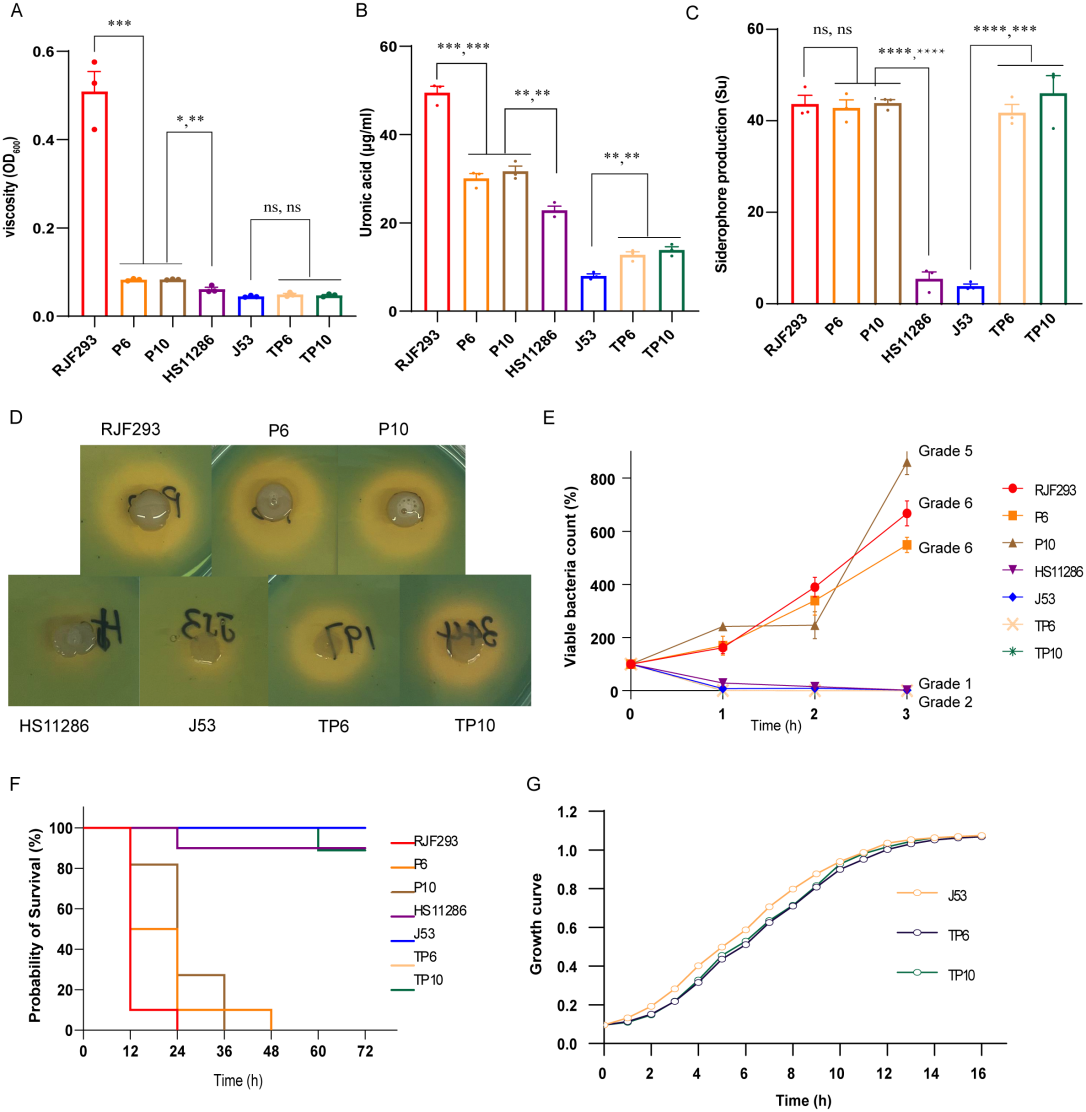
Virulence phenotypes of P6, P10, and their transconjugants. **(A)** Growth curves of P6, P10, and their transconjugants. **(B)** Viscosity measurements. **(C)** CPS production assays. **(D)** Siderophore production assessed using CAS agar. **(E)** Quantitative measurement of siderophore production. **(F)** Serum killing assay results. **(G)** Survival curves of *Galleria mellonella* larvae infected with the indicated strains. ****P< 0.0001, ***P< 0.001, **P< 0.01, *P< 0.05, ns, not significant.

P6, P10 displayed multidrug-resistant (MDR) against various antimicrobial agents (**Table 2**), consistent with the presence of *bla*_KPC-2_, *bla*_TEM-1_, *aac(2)-IId*, *bla*_CTX-M-15_ (**Table 2**). Acquisition of virulence plasmids imposed measurable fitness costs (**Figure 5A**). Both strains produced less capsule polysaccharide (CPS) and exhibited lower viscosity than the hypervirulent positive control strain RJF293 (K2-ST65), but more than that of the less-virulent negative control strain HS11286 (K64-ST11) (**Figures 5B, C**). Siderophore production of P6 and P10 was approximately 9-fold higher than in HS11286 and approached RJF293 levels (**Figures 5D, E**). Serum-killing assays confirmed their high virulence, with robust serum resistance (Grade 5 and Grade 6) (**Figure 5F**), and this was further validated in animal models (**Figure 5G**). Thus, *K. pneumoniae* P6 and P10 exhibited both carbapenem resistance and hypervirulence. However, the KPC plasmid was rapidly lost in both P6 and P10 strains (**Supplementary Figure S4**), suggesting that ST65-CR-hvKp strains may struggle to maintain both carbapenem resistance and hypervirulence simultaneously. While the 10th generation P6 strain (P6-10th) retained similar virulence and resistance profiles, the KPC lost-derivative (P6-10th-KPClost) became carbapenem-sensitive (**Supplementary Figure S3** and **Supplementary Table S3**).

**Table 2:** Antimicrobial susceptibilities of strains and their transconjugants.

Strains	Bacterial species	MIC (µg/mL)^a^															
		AMP	SAM	TZP	CXM	CRO	FEP	CHL	GEN	LVX	SXT	IPM	MEM	TGC	NIT	AMK	COL
P6	*K. pneumoniae*	–	>64/32^R^	>256/4^R^	>64^R^	>8^R^	64^R^	8^S^	16^R^	<0.5^S^	<0.25/4.75^S^	32^R^	64^R^	<0.5^S^	64^I^	32^R^	<0.5^S^
P10	*K. pneumoniae*	–	>64/32^R^	>256/4^R^	>64^R^	>8^R^	>64^R^	8^S^	16^R^	<0.5^S^	<0.25/4.75^R^	64^R^	64^R^	<0.5^S^	64^I^	32^R^	<0.5^S^
J53	*E. coli*	>2^S^	4/2^S^	<8/4^S^	4^S^	<0.25^S^	4^S^	8^S^	<1^S^	<0.5^S^	<0.25/4.75^S^	<0.25^S^	<0.25^S^	<0.5^S^	16^S^	<8^S^	<0.5^S^
Transconjugants																	
TP6-1	*E. coli*	>64^S^	>64/32^R^	<8/4^S^	>64^R^	<0.25^S^	<2^S^	4^S^	16^R^	<0.5^S^	<0.25/4.75^S^	<0.25^S^	<0.25^S^	<0.5^S^	<8^S^	32^R^	<0.5^S^
TP6-2	*E. coli*	>64^S^	>64/32^R^	<8/4^S^	>64^R^	<0.25^S^	<2^S^	4^S^	8^I^	<0.5^S^	<0.25/4.75^S^	<0.25^S^	<0.25^S^	<0.5^S^	16^S^	32^R^	<0.5^S^
TP6-3	*E. coli*	>64^S^	>64/32^R^	<8/4^S^	16^I^	<0.25^S^	<2^S^	8^S^	16^R^	<0.5^S^	<0.25/4.75^S^	<0.25^S^	<0.25^S^	<0.5^S^	32^S^	32^R^	<0.5^S^
TP10-1	*E. coli*	>64^S^	>64/32^R^	<8/4^S^	>64^R^	>8^R^	64^R^	8^S^	16^R^	<0.5^S^	<0.25/4.75^S^	<0.25^S^	<0.25^S^	<0.5^S^	128^R^	<8^S^	<0.5^S^
TP10-2	*E. coli*	>64^S^	32/16^R^	<8/4^S^	>64^R^	>8^R^	32^R^	8^S^	16^R^	<0.5^S^	<0.25/4.75^S^	<0.25^S^	<0.25^S^	<0.5^S^	64^I^	<8^S^	<0.5^S^
TP10-3	*E. coli*	>64^S^	32/16^R^	<8/4^S^	>64^R^	>8^R^	32^R^	8^S^	16^R^	<0.5^S^	<0.25/4.75^S^	<0.25^S^	<0.25^S^	<0.5^S^	64^I^	<8^S^	<0.5^S^

a AMP, ampicillin; SAM, ampicillin/sulbactam; TZP, piperacillin/tazobactam; CXM, cefuroxime; CRO, ceftriaxone; FEP, cefepime; CHL, chloramphenicol; GEN, gentamicin; LVX, levofloxacin; SXT, trimethoprim/sulphamethoxazole; IPM, imipenem; MEM, meropenem; TGC, tigecycline; NIT, nitrofurantion. AMK, amikacin; COL, colistin.

Transconjugants that acquired plasmids from P6 and P10 showed increased resistance to certain antimicrobial agents but remained carbapenem-sensitive as the *bla*_KPC-2_-carrying pP3 was not co-transferred (**Table 2**). Acquisition of the virulence plasmid from P6 and P10 significantly increased uronic acid levels and siderophore production, although viscosity, serum resistance, and larval mortality rates remained comparable to *E. coli* J53 (**Figure 5**).

These findings demonstrate that virulence plasmid transfer can simultaneously disseminate resistance and virulence traits, reshaping the phenotypic landscape of recipient bacteria.

## 4 Discussion

Infections caused by CR-hvKp strains pose a significant global threat to human health, with repercussions exceeding those of either individual hvKp or CRKP infections alone (Gu et al., 2018). Previous studies have highlighted the ongoing expansion of convergent *K. pneumoniae* strains, with hv-CRKP strains (particularly ST11) being the most prevalent (Tian et al., 2022; Yang et al., 2022). However, CR-hvKp strains (typified by ST23 and ST65) also warrant close attention. A PubMed search using the terms “ST65”, “carbapenem-resistant”, “hypervirulent”, and “*Klebsiella*” retrieved only 10 records, highlighting the scarcity of clinical reports despite the considerable pathogenicity and multidrug resistance associated with these strains.

ST65 *K. pneumoniae* belongs to the K2 capsular type associated with CPS overproduction (Jun, 2018). Traditionally, ST65 has been linked to classical hvKp carrying non-conjugative pLVPK-like virulence plasmids lacking resistance genes (Bialek-Davenet et al., 2014; Wyres et al., 2019). Our previous research demonstrated that when ST65 strain acquired a KPC plasmid *in vitro*, it was unable to maintain carbapenem-resistance and CPS overproduction simultaneously, often leading to mutations in capsular biosynthesis genes (Tian et al., 2022). Both capsule synthesis and *bla*_KPC_ expression are energetically demanding processes that impose significant metabolic costs on the bacterium (Cortina et al., 2018; Whitfield et al., 2020). In this study, we identified two clinical ST65 *K. pneumoniae* strains exhibiting both carbapenem resistance and hypervirulence; however, these traits appear to be unstable, which may explain the lower prevalence of ST65-CR-hvKp compared to ST11 hv-CRKP strains.

Our findings also shed light on the mechanisms facilitating the transfer of non-conjugative virulence plasmids (Li et al., 2020; Xie et al., 2020; Zhao et al., 2022). While previous studies have demonstrated the role of crossover recombination and Tn*3*-mediated replicative transposition in fusing KPC and virulence plasmids (Xie et al., 2020; Zhao et al., 2022; Tian et al., 2022), our results reveal a novel IS*26*-mediated hybrid plasmid formation in ST65 CR-hvKp. IS*26* is well known to facilitate plasmid rearrangement and does not transpose independently; instead, it forms cointegrates as the final products of IS*26* transposase activity (Harmer and Hall, 2016; 2021). Mobile genetic elements (MGEs), such as transposons, insertion sequences (IS), are key drivers of resistance gene dissemination and bacterial adaptation (Partridge et al., 2018; Tang et al., 2022).

Recent studies have shown that non-conjugative pLVPK-like virulence plasmids can be mobilized by conjugative *bla*_KPC_-positive IncFII plasmids (Xu et al., 2021; Tian et al., 2022). These conjugative plasmids encode the machinery and factors required to transfer genetic material from one bacterial cell to another, even when the virulence plasmid itself cannot transfer autonomously. In our study, the untyped KPC plasmid in ST65 strains encoded a complete *vir-*type T4SS, while an additional conjugative IncM2 plasmid encoded a *tra-trb*-type T4SS. Interestingly, we found that it was the conjugative IncM2 plasmid, not the KPC plasmid, which mobilized the non-conjugative virulence plasmid. This finding expands current understanding of the intricate molecular mechanisms underlying virulence plasmid transfer. Furthermore, we observed that CPS and siderophore production were significantly elevated in recipient strains following acquisition of the virulence plasmid, although serum resistance and *Galleria. mellonella* mortality did not differ significantly. This aligns with previous studies indicating that virulence in *K. pneumoniae* is multifactorial, and that acquisition of a single plasmid may not be sufficient to dramatically alter virulence phenotypes (Tian et al., 2021; Zhu et al., 2021; Hu et al., 2023).

The dissemination of virulence plasmids plays a central role in CR-hvKp evolution and represents a growing clinical challenge. Although these strains may only transiently sustain both hypervirulence and carbapenem resistance, they serve as critical intermediates for virulence plasmid transmission. Our findings suggest that helper plasmids responsible for mobilizing non-conjugative virulence plasmids are not limited to the *bla*_KPC_-positive types. Various conjugative plasmids may participate in the transfer of virulence determinants, and the presence of *oriT* sequences similar to those of helper conjugative plasmids emerges as critical for relaxase recognition and initiation of the conjugation (O’Brien et al., 2015; Xu et al., 2021). However, further investigation is needed to determine which specific plasmids can mobilize pLVPK-like virulence plasmids.

There are several limitations to this study. The scarcity of ST65-CR-hvKp strains in clinical settings limited our analysis to only two isolates included, emphasizing the need for larger studies. Additionally, potential plasmid recombination events during conjugation may not have been fully captured, underscoring the importance of incorporating a broader collection of clinically sequenced *K. pneumoniae* genomes in future investigations.

In conclusion, we reveal two novel transmission mechanisms of virulence plasmids in ST65 CR-hvKp strains: 1) IncM2 plasmids can mobilize virulence plasmid; 2) IncM2 plasmids facilitate virulence plasmid transfer via recombination with virulence plasmids through IS*26* sequences. The virulence and resistance traits can also be transferred through virulence and resistance plasmid. Given the therapeutic challenges posed by CR-hvKp, implementing strict infection control measures is imperative.

## Data Availability

The datasets presented in this study can be found in online repositories. The names of the repository/repositories and accession number(s) can be found in the article/**Supplementary Material**.

## References

[B1] Al Bshabshe A. Al-Hakami A. Alshehri B. Al-Shahrani K. A. Alshehri A. A. Al Shahrani M. B.. (2020). Rising klebsiella pneumoniae infections and its expanding drug resistance in the intensive care unit of a tertiary healthcare hospital, Saudi Arabia. Cureus 12, e10060. doi: 10.7759/cureus.10060 32999783 PMC7520404

[B2] Bialek-Davenet S. Criscuolo A. Ailloud F. Passet V. Jones L. Delannoy-Vieillard A. S.. (2014). Genomic definition of hypervirulent and multidrug-resistant Klebsiella pneumoniae clonal groups. Emerg. Infect. Dis. 20, 1812–1820. doi: 10.3201/eid2011.140206 25341126 PMC4214299

[B3] Borgmann S. Pfeifer Y. Becker L. Rieß B. Siegmund R. Sagel U. (2018). Findings from an outbreak of carbapenem-resistant Klebsiella pneumoniae emphasize the role of antibiotic treatment for cross transmission. Infection 46, 103–112. doi: 10.1007/s15010-017-1103-3 29177610

[B4] Chin C. S. Peluso P. Sedlazeck F. J. Nattestad M. Concepcion G. T. Clum A.. (2016). Phased diploid genome assembly with single-molecule real-time sequencing. Nat. Methods 13, 1050–1054. doi: 10.1038/nmeth.4035 27749838 PMC5503144

[B5] Choby J. E. Howard-Anderson J. Weiss D. S. (2020). Hypervirulent Klebsiella pneumoniae – clinical and molecular perspectives. J. Internal Med. 287, 283–300. doi: 10.1111/joim.13007 31677303 PMC7057273

[B6] Cortina G. A. Hays J. M. Kasson P. M. (2018). Conformational intermediate that controls KPC-2 catalysis and beta-lactam drug resistance. ACS Catal 8, 2741–2747. doi: 10.1021/acscatal.7b03832 30637173 PMC6324736

[B7] Gu D. Dong N. Zheng Z. Lin D. Huang M. Wang L.. (2018). A fatal outbreak of ST11 carbapenem-resistant hypervirulent Klebsiella pneumoniae in a Chinese hospital: a molecular epidemiological study. Lancet Infect. Dis. 18, 37–46. doi: 10.1016/s1473-3099(17)30489-9 28864030

[B8] Guo M. Q. Wang Y. T. Wang S. S. Chen L. K. Xu Y. H. Li G. (2024). Genomic epidemiology of hypervirulent carbapenem-resistant Klebsiella pneumoniae at Jinshan local hospital, Shanghai, during 2014-2018. J. Microbiol. Immunol. Infect. 57, 128–137. doi: 10.1016/j.jmii.2023.10.012 37951801

[B9] Han Y. L. Wen X. H. Zhao W. Cao X. S. Wen J. X. Wang J. R.. (2022). Epidemiological characteristics and molecular evolution mechanisms of carbapenem-resistant hypervirulent Klebsiella pneumoniae. Front. Microbiol. 13. doi: 10.3389/fmicb.2022.1003783 PMC9524375 36188002

[B10] Hao M. He Y. Zhang H. Liao X. P. Liu Y. H. Sun J.. (2020). CRISPR-cas9-mediated carbapenemase gene and plasmid curing in carbapenem-resistant enterobacteriaceae. Antimicrob. Agents Chemother. 64, e00843-20. doi: 10.1128/aac.00843-20 32631827 PMC7449206

[B11] Harmer C. J. Hall R. M. (2016). IS26-mediated formation of transposons carrying antibiotic resistance genes. mSphere 1, 00038-16. doi: 10.1128/mSphere.00038-16 PMC4894685 27303727

[B12] Harmer C. J. Hall R. M. (2021). IS26 cannot move alone. J. Antimicrob. Chemother. 76, 1428–1432. doi: 10.1093/jac/dkab055 33686401

[B13] Hu D. Chen W. Wu J. Luo X. Yu L. Qu Y.. (2023). Coexistence of c-rmpA with p-rmpA and p-rmpA2 rather than excessive siderophores confers higher virulence in K1 Klebsiella pneumoniae. Pathology 55, 1004–1012. doi: 10.1016/j.pathol.2023.07.007 37802741

[B14] Jensen C. S. Iversen K. H. Dargis R. Shewmaker P. Rasmussen S. Christensen J. J.. (2021). Streptococcus pseudopneumoniae: use of whole-genome sequences to validate species identification methods. J. Clin. Microbiol. 59, 02503-20. doi: 10.1128/jcm.02503-20 PMC8111133 33208473

[B15] Jun J. B. (2018). Klebsiella pneumoniae liver abscess. Infect. Chemother. 50, 210–218. doi: 10.3947/ic.2018.50.3.210 30270580 PMC6167513

[B16] Kaas R. S. Leekitcharoenphon P. Aarestrup F. M. Lund O. (2014). Solving the problem of comparing whole bacterial genomes across different sequencing platforms. PloS One 9, e104984. doi: 10.1371/journal.pone.0104984 25110940 PMC4128722

[B17] Koren S. Walenz B. P. Berlin K. Miller J. R. Bergman N. H. Phillippy A. M. (2017). Canu: scalable and accurate long-read assembly via adaptive k-mer weighting and repeat separation. Genome Res. 27, 722–736. doi: 10.1101/gr.215087.116 28298431 PMC5411767

[B18] Lam M. M. C. Wyres K. L. Duchene S. Wick R. R. Judd L. M. Gan Y. H.. (2018). Population genomics of hypervirulent Klebsiella pneumoniae clonal-group 23 reveals early emergence and rapid global dissemination. Nat. Commun. 9, 2703. doi: 10.1038/s41467-018-05114-7 30006589 PMC6045662

[B19] Li R. Cheng J. Dong H. Li L. Liu W. Zhang C.. (2020). Emergence of a novel conjugative hybrid virulence multidrug-resistant plasmid in extensively drug-resistant Klebsiella pneumoniae ST15. Int. J. Antimicrob. Agents 55, 105952. doi: 10.1016/j.ijantimicag.2020.105952 32335274

[B20] Martin R. M. Bachman M. A. (2018). Colonization, infection, and the accessory genome of klebsiella pneumoniae. Front. Cell. Infection Microbiol. 8. doi: 10.3389/fcimb.2018.00004 PMC5786545 29404282

[B21] O’Brien F. G. Yui Eto K. Murphy R. J. Fairhurst H. M. Coombs G. W. Grubb W. B.. (2015). Origin-of-transfer sequences facilitate mobilisation of non-conjugative antimicrobial-resistance plasmids in Staphylococcus aureus. Nucleic Acids Res. 43, 7971–7983. doi: 10.1093/nar/gkv755 26243776 PMC4652767

[B22] Partridge S. R. Kwong S. M. Firth N. Jensen S. O. (2018). Mobile genetic elements associated with antimicrobial resistance. Clin. Microbiol. Rev. 31, 00088-17. doi: 10.1128/cmr.00088-17 PMC6148190 30068738

[B23] Pu D. Zhao J. Chang K. Zhuo X. Cao B. (2023). Superbugs” with hypervirulence and carbapenem resistance in Klebsiella pneumoniae: the rise of such emerging nosocomial pathogens in China. Sci. Bull. (Beijing) 68, 2658–2670. doi: 10.1016/j.scib.2023.09.040 37821268

[B24] Sahly H. Aucken H. Benedí V. J. Forestier C. Fussing V. Hansen D. S.. (2004). Increased serum resistance in Klebsiella pneumoniae strains producing extended-spectrum beta-lactamases. Antimicrob. Agents Chemother. 48, 3477–3482. doi: 10.1128/aac.48.9.3477-3482.2004 15328114 PMC514775

[B25] Tamma P. D. Aitken S. L. Bonomo R. A. Mathers A. J. van Duin D. Clancy C. J. (2023). Infectious diseases society of America 2023 guidance on the treatment of antimicrobial resistant gram-negative infections. Clin. Infect. Dis. ciad428. doi: 10.1093/cid/ciad428 37463564

[B26] Tang Y. Li G. Shen P. Zhang Y. Jiang X. (2022). Replicative transposition contributes to the evolution and dissemination of KPC-2-producing plasmid in Enterobacterales. Emerg. Microbes Infect. 11, 113–122. doi: 10.1080/22221751.2021.2013105 34846275 PMC8725868

[B27] Tian D. Liu X. Chen W. Zhou Y. Hu D. Wang W.. (2022). Prevalence of hypervirulent and carbapenem-resistant Klebsiella pneumoniae under divergent evolutionary patterns. Emerg. Microbes Infect. 11, 1936–1949. doi: 10.1080/22221751.2022.2103454 35844192 PMC9359173

[B28] Tian D. Wang B. Zhang H. Pan F. Wang C. Shi Y.. (2020). Dissemination of the bla (NDM-5) Gene via IncX3-Type Plasmid among Enterobacteriaceae in Children. mSphere 5, 00699-19. doi: 10.1128/mSphere.00699-19 PMC6952193 31915216

[B29] Tian D. Wang W. Li M. Chen W. Zhou Y. Huang Y.. (2021). Acquisition of the conjugative virulence plasmid from a CG23 hypervirulent klebsiella pneumoniae strain enhances bacterial virulence. Front. Cell. Infection Microbiol. 11. doi: 10.3389/fcimb.2021.752011 PMC8485780 34604119

[B30] Walker B. J. Abeel T. Shea T. Priest M. Abouelliel A. Sakthikumar S.. (2014). Pilon: an integrated tool for comprehensive microbial variant detection and genome assembly improvement. PloS One 9, e112963. doi: 10.1371/journal.pone.0112963 25409509 PMC4237348

[B31] Wang X. Xie Y. Li G. Liu J. Li X. Tian L.. (2018). Whole-Genome-Sequencing characterization of bloodstream infection-causing hypervirulent Klebsiella pneumoniae of capsular serotype K2 and ST374. Virulence 9, 510–521. doi: 10.1080/21505594.2017.1421894 29338592 PMC5955473

[B32] Whitfield C. Wear S. S. Sande C. (2020). Assembly of bacterial capsular polysaccharides and exopolysaccharides. Annu. Rev. Microbiol. 74, 521–543. doi: 10.1146/annurev-micro-011420-075607 32680453

[B33] Wyres K. L. Wick R. R. Judd L. M. Froumine R. Tokolyi A. Gorrie C. L.. (2019). Distinct evolutionary dynamics of horizontal gene transfer in drug resistant and virulent clones of Klebsiella pneumoniae. PloS Genet. 15, e1008114. doi: 10.1371/journal.pgen.1008114 30986243 PMC6483277

[B34] Xie M. Chen K. Ye L. Yang X. Xu Q. Yang C.. (2020). Conjugation of Virulence Plasmid in Clinical Klebsiella pneumoniae Strains through Formation of a Fusion Plasmid. Adv. Biosyst. 4, e1900239. doi: 10.1002/adbi.201900239 32293159

[B35] Xu Y. Zhang J. Wang M. Liu M. Liu G. Qu H.. (2021). Mobilization of the nonconjugative virulence plasmid from hypervirulent Klebsiella pneumoniae. Genome Med. 13, 119. doi: 10.1186/s13073-021-00936-5 34294113 PMC8299605

[B36] Yang X. Sun Q. Li J. Jiang Y. Li Y. Lin J.. (2022). Molecular epidemiology of carbapenem-resistant hypervirulent Klebsiella pneumoniae in China. Emerg. Microbes Infect. 11, 841–849. doi: 10.1080/22221751.2022.2049458 35236251 PMC8942559

[B37] Zhao Q. Feng Y. Zong Z. (2022). Conjugation of a hybrid plasmid encoding hypervirulence and carbapenem resistance in klebsiella pneumoniae of sequence type 592. Front. Microbiol. 13. doi: 10.3389/fmicb.2022.852596 PMC9085563 35558122

[B38] Zhou Y. Tang Y. Fu P. Tian D. Yu L. Huang Y.. (2020). The type I-E CRISPR-Cas system influences the acquisition of blaKPC-IncF plasmid in Klebsiella pneumonia. Emerg. Microbes Infect. 9, 1011–1022. doi: 10.1080/22221751.2020.1763209 32393110 PMC7301723

[B39] Zhu J. Wang T. Chen L. Du H. (2021). Virulence factors in hypervirulent klebsiella pneumoniae. Front. Microbiol. 12. doi: 10.3389/fmicb.2021.642484 PMC8060575 33897652

