# Supplementary material for: Genomic evolution and dissemination of non-conjugative virulence plasmid of ST65 carbapenem-resistant and hypervirulent Klebsiella pneumoniae strains in a Chinese hospital
Source: Front Cell Infect Microbiol. 2025 Jun 12;15:1548300. doi: 10.3389/fcimb.2025.1548300 (PMC12198223; doi:10.3389/fcimb.2025.1548300)
Supplement: Supplementary file 2 [file SupplementaryFile1.docx]

**Supplementary Materials**


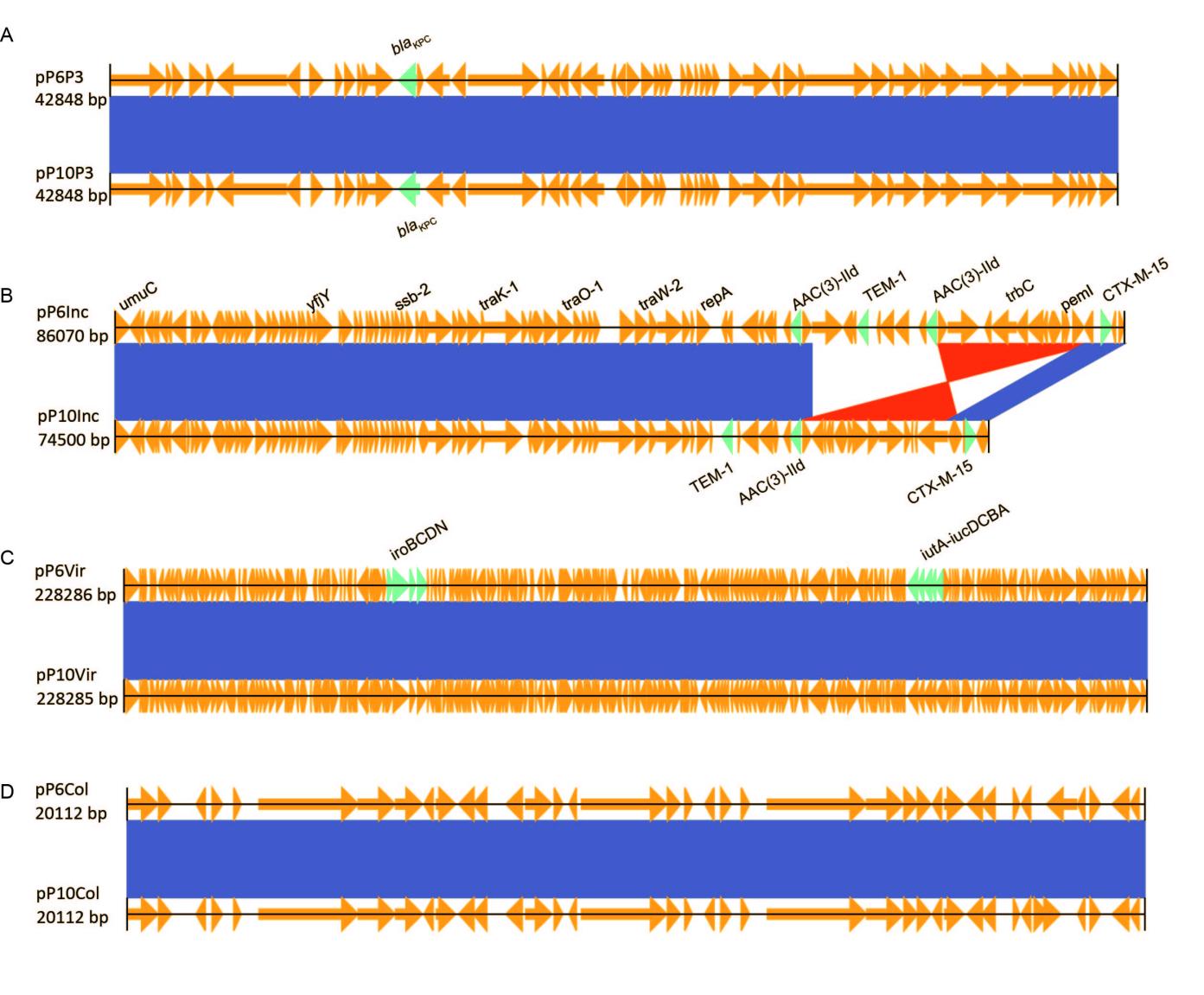


**Figure S1. Plasmid comparison of P6 and P10.** (A) Comparison of pP6P3 and pP10P3; (B) Comparison of pP6Inc and pP10Inc; (C) Comparison of pP6Vir and pP10Vir; (D) Comparison of pP6Col and pP10Col.

**
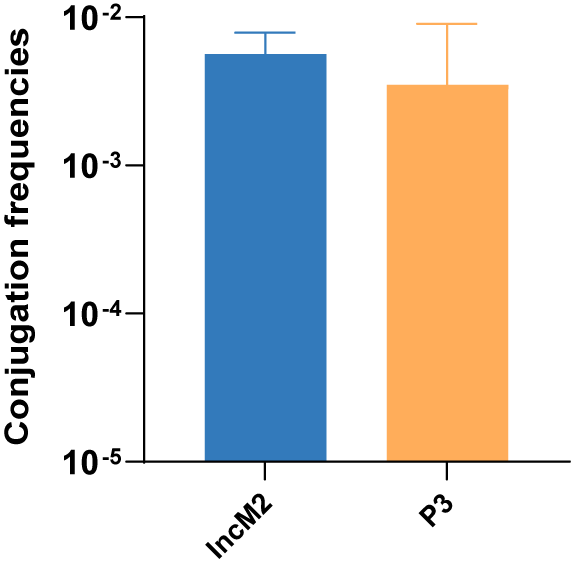
Figure S2. The conjugative frequencies of IncM2 plasmid and KPC plasmid (P3).**

**
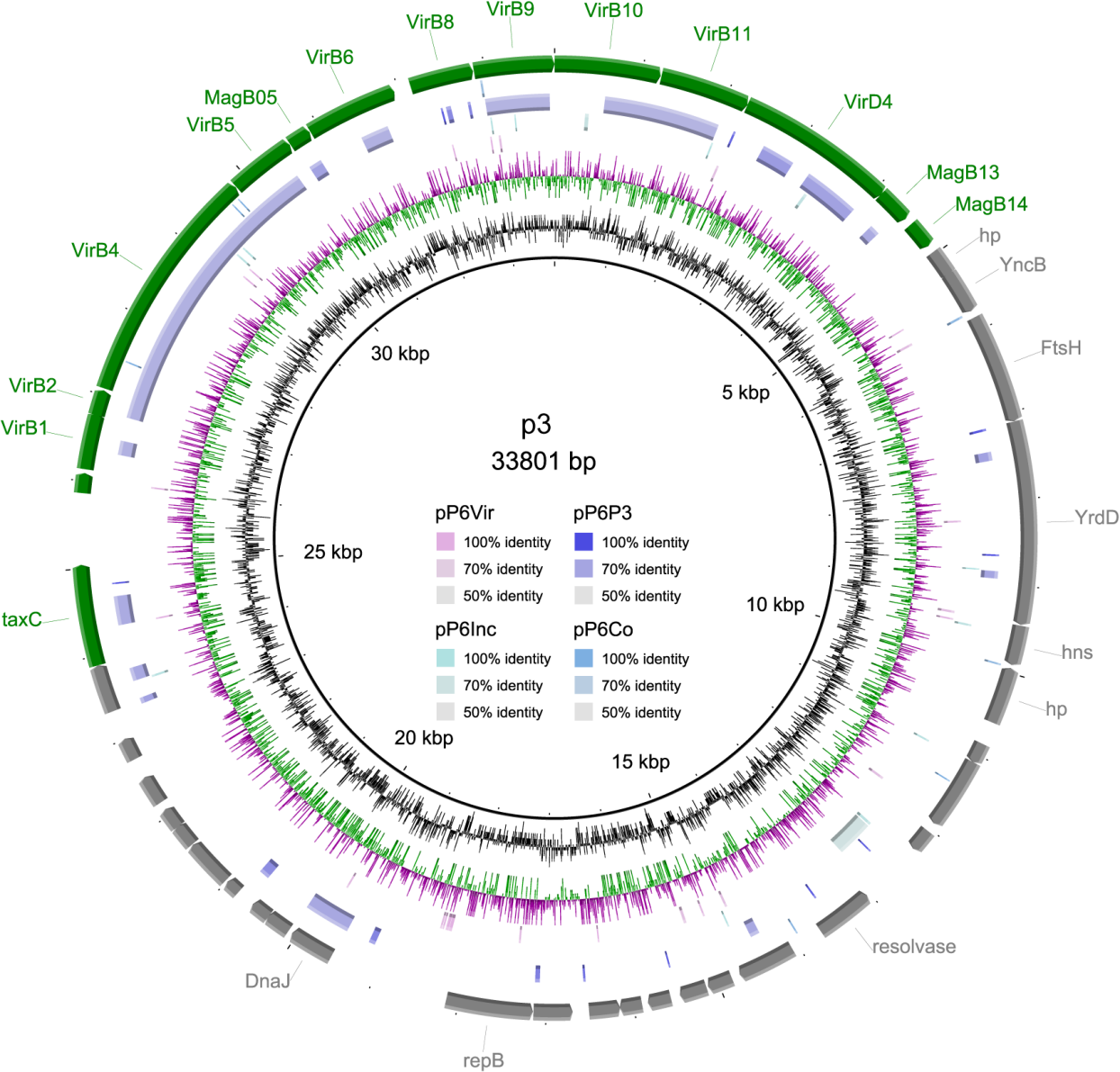
**

**Figure S3. Comparative genome analysis of p3 plasmid.** The pP6vir, pP6P3, pP6Inc, and pP6Co plasmids were compared to the reference genome p3 using BRIG software to explore the origin of p3 plasmid emerged in transconjugants.


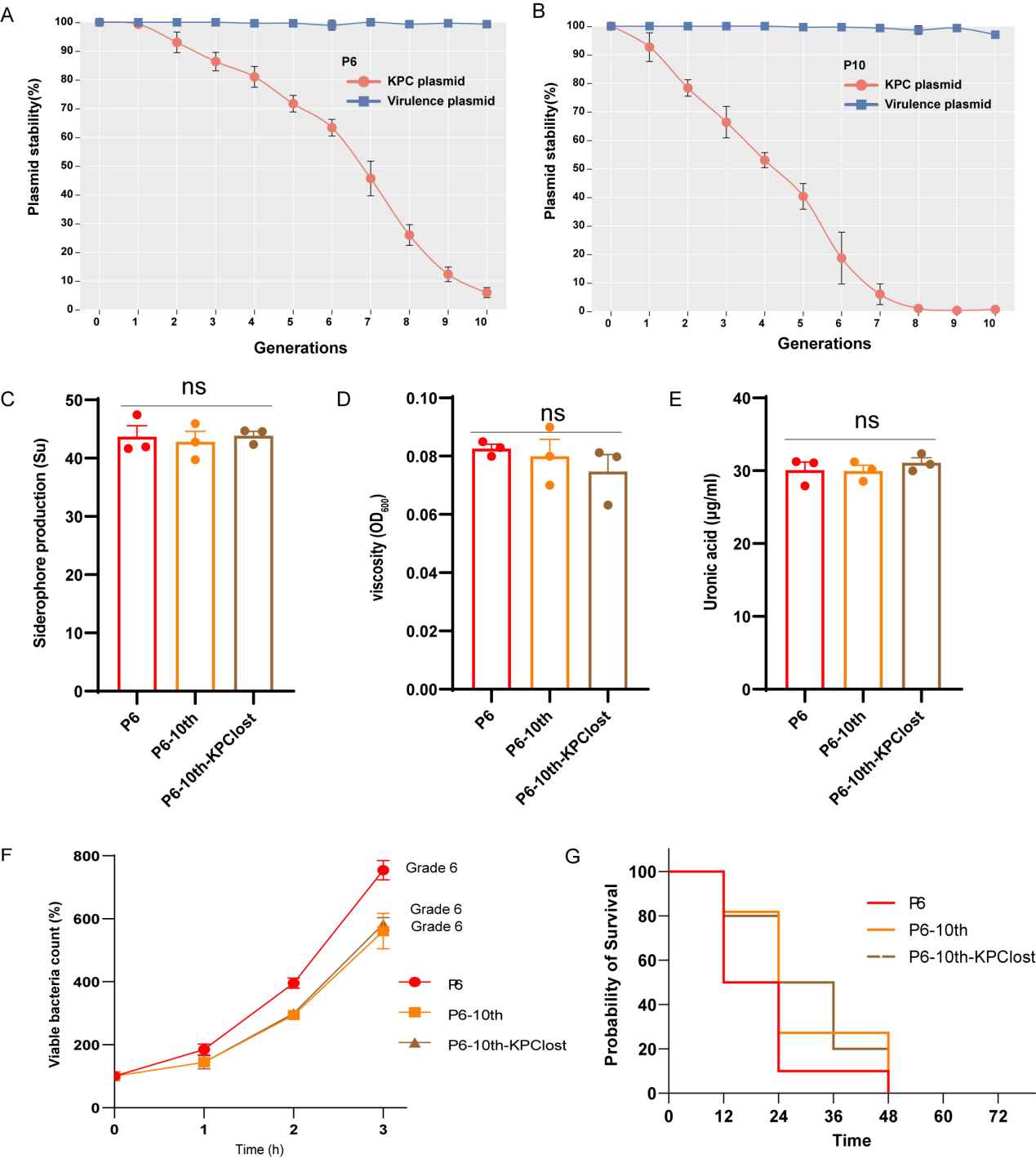


**Figure S4. Virulence phenotypes of P6 and 10th generation P6 strains.** (A) Plasmid stabilities of P6. (B) Plasmid stabilities of P10. (C) Siderophore production. (D) Viscosity. (E) Uronic acid. (F) Serum resistance. (G) The survival curves of infected Larvae wax.

**Table S1 Primers used in this study**

| **Name** | **Sequences (5’-3’)** |
| --- | --- |
| **For screening transconjugants** | |
| iucA-F | GCTTATTTCTCCCCAACCC |
| iucA-R | TCAGCCCTTTAGCGACAAG |
| oqxA-F | CCAAAGTGACCGCCCCTATT |
| oqxA-R | GACGATGACGCTATCCCCAG |
| YeeU-F | ACGCTGAGTGGGGTAAATGG |
| YeeU-R | TGCAGCGGGAGATTACACTG |
| **For constructing of the pCascure plasmids** | |
| SpeI-N20IncM-gRNA-F | AATACTAGT-TCAACGGTGCTGACCAGGAG-GTTTTAGAGCTAGAAATAGC |
| XbaI-gRNA-R | CTGGTTTCTAGAAGTAGTGGA |
| SpeI-N20P3-gRNA-F | AATACTAGT-TGGAAGTCACGTTACCTGA-TGTTTTAGAGCTAGAAATAGC |
| **For screening plasmid-cured strains** | |
| pVir gene-F | TGGGGAAGGCTCTCGATGTA |
| pVir gene-R | CCAGGAGTAAGGCAAACCGT |
| pInc gene-F | GCGCGTTAAGTCTGCGTATG |
| pInc gene-R | ACTCGCTGGGTAAACTGCTC |
| pP3 gene-F | CCTTTCGGGATGAACTCGCT |
| pP3 gene-R | GTGTACCAGCCAGCCTTTCA |

**Table S2** **Plasmids of TP6 and TP10 transconjugants.**

| **Strains** | **Plasmids** | **Accession no.** | **Incompatibility group** | **Length (bp)** | **Resistance factors** | **Virulence factors** |
| --- | --- | --- | --- | --- | --- | --- |
| TP6-1 | TP6-1-p1 | CP139678.1 | IncFIB_K_/IncHI1B | 229105 | - | *iucABCD-iutA; iroBCD-iroN; rmpA; rmpA2* |
|  | TP6-1-p2 | CP139679.1 | Untypeable | 33801 | - |  |
|  | TP6-1-p3 | CP139680.1 | ColRNAI | 19459 | *bla*_TEM-1_; *aac(3)-IId* |  |
| TP6-2 | TP6-2-p1 | CP139674.1 | IncFIB_K_/IncHI1B | 229105 | - | *iucABCD-iutA; iroBCD-iroN; rmpA; rmpA2* |
|  | TP6-2-p2 | CP139675.1 | Untypeable | 33801 | - |  |
|  | TP6-2-p3 | CP139676.1 | ColRNAI | 19459 | *bla*_TEM-1_; *aac(3)-IId* |  |
| TP6-3 | TP6-3-p1 | CP139670.1 | IncFIB_K_/IncHI1B | 229113 | - | *iucABCD-iutA; iroBCD-iroN; rmpA; rmpA2* |
|  | TP6-3-p2 | CP139671.1 | Untypeable | 33801 | - |  |
|  | TP6-3-p3 | CP139672.1 | ColRNAI | 19459 | *bla*_TEM-1_; *aac(3)-IId* |  |
| TP10-1 | TP10-1-p1 | CP139666.1 | IncFIB_K_/IncHI1B/IncM2 | 303604 | *bla*_CTX-M-15_; *bla*_TEM-1_; *aac(3)-IId* | *iucABCD-iutA; iroBCD-iroN; rmpA; rmpA2* |
|  | TP10-1-p2 | CP139667.1 | Untypeable | 33801 | - |  |
|  | TP10-1-p3 | CP139668.1 | ColRNAI | 10046 | - |  |
| TP10-2 | TP10-2-p1 | CP139662.1 | IncFIB_K_/IncHI1B/IncM2 | 303604 | *bla*_CTX-M-15_; *bla*_TEM-1_; *aac(3)-IId* | *iucABCD-iutA; iroBCD-iroN; rmpA; rmpA2* |
|  | TP10-2-p2 | CP139663.1 | Untypeable | 33801 | - |  |
|  | TP10-2-p3 | CP139664.1 | ColRNAI | 10046 | - |  |
| TP10-3 | TP10-3-p1 | CP139658.1 | IncFIB_K_/IncHI1B/IncM2 | 303612 | *bla*_CTX-M-15_; *bla*_TEM-1_; *aac(3)-IId* | *iucABCD-iutA; iroBCD-iroN; rmpA; rmpA2* |
|  | TP10-3-p2 | CP139659.1 | Untypeable | 33801 | - |  |
|  | TP10-3-p3 | CP139660.1 | ColRNAI | 10046 | - |  |

**Table S3. Antimicrobial susceptibilities of P6 and their 10^th^ generation strains**

| **Strains** | **Bacterial**  **species** | **MIC (µg/mL)^a^** | | | | | | | | | | | | | | |
| --- | --- | --- | --- | --- | --- | --- | --- | --- | --- | --- | --- | --- | --- | --- | --- | --- |
|  |  | **SAM** | **TZP** | **CXM** | **CRO** | **FEP** | **CHL** | **GEN** | **LVX** | **SXT** | **IPM** | **MEM** | **TGC** | **NIT** | **AMK** | **COL** |
| P6 | *K. pneumoniae* | >64/32^R^ | >256/4^R^ | >64^R^ | >8^R^ | 64^R^ | 8^S^ | 16^R^ | <0.5^S^ | <0.25/4.75^S^ | 32^R^ | 64^R^ | <0.5^S^ | 64^I^ | 32^R^ | <0.5^S^ |
| P6-10th | *K. pneumoniae* | >64/32^R^ | >256/4^R^ | >64^R^ | >8^R^ | 64^R^ | 8^S^ | 16^R^ | <0.5^S^ | <0.25/4.75^R^ | 64^R^ | 64^R^ | <0.5^S^ | 64^I^ | 32^R^ | <0.5^S^ |
| P6-10th-KPClost | *K. pneumoniae* | >64/32^R^ | >256/4^R^ | >64^R^ | >8^R^ | 32^R^ | 8^S^ | 16^R^ | <0.5^S^ | <0.25/4.75^S^ | <0.25^S^ | <0.25^S^ | <0.5^S^ | 64^I^ | 32^R^ | <0.5^S^ |

^a^SAM, ampicillin/sulbactam; TZP, piperacillin/tazobactam; CXM, cefuroxime; CRO, ceftriaxone; FEP, cefepime; CHL, chloramphenicol; GEN, gentamicin; LVX, levofloxacin; SXT, trimethoprim/sulphamethoxazole; IPM, imipenem; MEM, meropenem; TGC, tigecycline; NIT, nitrofurantion.AMK, amikacin;COL, colistin.
